# Translocation of neuronal nitric oxide synthase to the plasma membrane by ATP is mediated by P2X and P2Y receptors

**DOI:** 10.1186/1744-8069-5-40

**Published:** 2009-07-20

**Authors:** Takayuki Ohnishi, Shinji Matsumura, Seiji Ito

**Affiliations:** 1 Department of Medical Chemistry, Kansai Medical University, 10-15 Fumizono, Moriguchi 570-8506, Japan; 2 Current address : Division of Pharmacology, Molecular and Cellular Medicine, Niigata University, Graduate School of Medical and Dental Sciences, 1-757 Asahimachi-dori, Chuo-ku, Niigata, 951-8510, Japan

## Abstract

**Background:**

The translocation of neuronal nitric oxide synthase (nNOS) from the cytosol to the membrane is functionally coupled to the activation of *N*-methyl-D-aspartate (NMDA) receptors at synapses. Whereas there is abundant evidence indicating that ATP and nitric oxide are involved in nociceptive transmission, whether nNOS is activated by ATP remains unknown. We recently established a fluorescence imaging system for examining nNOS translocation in PC12 cells expressing a yellow fluorescence protein-tagged nNOS N-terminal mutant, nNOSNT-YFP, and examined the effect of ATP on nNOS translocation using the system.

**Results:**

The translocation of nNOS was induced by ATP in the presence of NMDA and forskolin, an adenylate cyclase activator. The purinergic P2X receptor agonist 2-MeSATP and the P2Y agonist UTP significantly enhanced nNOS translocation; and simultaneous stimulation with 2-MeSATP and UTP exhibited the same concentration-response curve for the translocation as obtained with ATP. ATP, 2-MeSATP, and UTP increased the intracellular Ca^2+ ^concentration ([Ca^2+^]i) in PC12 cells. Conversely, whereas the P2X receptor antagonist PPADS and the P2Y antagonist reactive blue-2 partially inhibited increases in the translocation of nNOS and [Ca^2+^]i by ATP, the non-selective P2 receptor antagonist suramin completely blocked them. In addition, the increase in the nNOS translocation by ATP was blocked by NMDA receptor antagonists and inhibitors of protein kinase A, protein kinase C, and Src kinase. Consistent with the expression of P2X and P2Y receptors in the spinal cord, ATP and UTP increased the [Ca^2+^]i in primary cultured spinal neurons. ATP potentiated and prolonged the [Ca^2+^]i increase produced by NMDA in the dorsal horn of the spinal cord. Furthermore, the selective P2X_3_/P2X_2/3 _antagonist A-317491 inhibited nNOS activation assessed by NO formation in spinal slices prepared from neuropathic pain model mice.

**Conclusion:**

ATP is involved in nNOS translocation mediated by protein kinase C via activation of P2X and P2Y receptors and nNOS translocation may be an action mechanism of ATP in nocieptive processing in the spinal cord.

## Background

Adenine and uridine nucleotides are present in tissues and released from all different types of cells in the nervous system as well as from damaged tissues in the periphery under pathophysiological conditions. The released nucleotides are implicated in diverse sensory processes including pain transmission via purinergic P2X and P2Y receptors [1,2]. To date 7 ionotropic P2X receptors [3] and 8 G-protein-coupled metabotropic P2Y receptors [4] have been cloned, and most of them are expressed on primary afferent neurons or spinal dorsal horn neurons. Exogenous administration of ATP and P2X-receptor agonists into the hind paw caused short-lasting nocifensor behaviors and thermal hyperalgesia [5,6], as well as relatively long-lasting mechanical allodynia [7], in rodents. On the other hand, P2 antagonists including A-317491, a selective P2X_3_/P2X_2/3_-receptor antagonist decreased various nociceptive behaviors, inflammatory hyperalgesia, and neuropathic pain [8-11]. P2X_3_-deficient mice have reduced pain-related behaviors in the formalin test [12]. Tsuda *et al*. also reported that the increased expression of P2X_4_-receptors induced by nerve injury or ATP stimulation in the spinal microglia produced allodynia [13].

In the central nervous system, nitric oxide (NO) is produced by neuronal NO synthase (nNOS) following the influx of Ca^2+ ^through *N*-methyl-D-aspartate (NMDA) receptors [14-16], and has been implicated in synaptic plasticity such as central sensitization in the spinal cord [17,18]. Co-localization of nNOS with NMDA receptors at the postsynaptic density (PSD) suggests that NMDA-receptor activity may be coupled to nNOS activation by a close spatial interaction [19]. We recently showed that the increase in nNOS activity in the superficial dorsal horn of the spinal cord reflects a neuropathic pain state even 1 week after nerve injury [20] and that this nNOS activation may be reversibly regulated by the translocation of nNOS from the cytosol to the plasma membrane in the presence of NMDA and the neuropeptide pituitary adenylate cyclase-activating polypeptide (PACAP) [21]. Unlike endothelial and inducible NOSs that anchor to the membrane by lipid modification, nNOS is unique in having an ~ 250 a.a. N-terminal extension containing a PSD-95/disc large/zonula occludens-1 (PDZ) domain and is recruited to membranes via protein-protein interactions [15,16]. We recently constructed a yellow fluorescence protein (YFP)-tagged nNOS N-terminal mutant encompassing amino acid residues 1–299 (nNOSNT-YFP) and succeeded in visualizing its translocation by co-stimulation with NMDA and PACAP in PC12 cells stably expressing it [22]. Thereby we demonstrated that PACAP was involved in nNOS translocation through the activation of both protein kinase C (PKC) following calcium mobilization and protein kinase A (PKA) mediated by PACAP receptor 1. ATP acts as an excitatory neurotransmitter in the dorsal horn of the spinal cord [23]. The activation of P2X receptors not only mediates but also facilitates excitatory transmission, releasing glutamate from primary afferent fibers in the spinal cord [24,25]. In the present study, we demonstrated that ATP could translocate nNOS from the cytosol to the plasma membrane mediated by PKC via activation of P2X and P2Y receptors in the presence of NMDA and forskolin, an adenylate cyclase activator, by using a fluorescence imaging system.

## Methods

### Materials

PC12 cells and PC12 cells stably expressing nNOSNT-YFP (PC12N cells) were maintained in Dulbecco's modified Eagle medium (DMEM) supplemented with 5% fetal calf serum, 10% horse serum, 50 U/ml penicillin, and 50 μg/ml streptomycin at 37°C in a 5% CO_2 _atmosphere. The chemicals used and their sources were as follow: NMDA, MK-801, 2-amino-5-phosphonovaleric acid (APV), calphostin C, PP2, suramin, pyridoxal-phosphate-6-azophenyl-2',4'-sulfonic acid (PPADS), 2-(methylthio) adenosine 5'-triphosphate (2-MeSATP), 2-(4-morpholinyl)-8-phenyl-4*H*-1-benzopyran- 4-one (LY294002), 2-(2-amino-3-methoxyphenyl)-4*H*-1-benzopyran-4-one (PD98059), reactive blue-2 (RB-2), A-317491, 4-(4-fluorophenyl)-2-(4-methylsulfinylphenyl)-5-(4-pyridyl)-1*H*-imidazole (SB203580), cytosine β-D-arabinofuranoside (Ara-C), Dnase I, and 8-bromo-cAMP (8-Br-cAMP) from Sigma (St. Louis, MO, USA); 8-Br-cGMP from Calbiochem (La Jolla, CA, USA); nerve growth factor (NGF), roscovitine, and UTP from Wako Pure Chemicals (Osaka, Japan); ATP from Oriental Yeast Co. (Tokyo, Japan); 1-[*N*, *O*-bis(5-isoquinolinesulfonyl)-*N*-methyl-L-tyrosyl]-4-phenylpiperazine (KN-62) and *N*-[2-(4-bromocinnamylamino)-ethyl]-5-isoquinoline (H-89) from Seikagaku Kogyo (Tokyo, Japan); fura-2 acetoxymethyl ester and *N*^G^-nitro-L-arginine methyl ester (L-NAME) from Dojindo (Kumamoto, Japan); diaminorhodamine-4M acetoxymethyl ester (DAR-4M AM) from Daiichi Pure Chemicals (Tokyo, Japan); and PACAP from Peptide Institute (Osaka, Japan). Other chemicals were of reagent grade.

Male ddY mice were purchased from Shizuoka Laboratory Centre (Hamamatsu, Japan). The animals were housed under conditions of a 12-h light-darkness cycle, a constant temperature of 22 ± 2°C and 60 ± 10% humidity. They were allowed free access to food and water before testing. All animal experiments were carried out in accordance with the National Institutes of Health guide for the care and use of laboratory animals and were approved by the Animal Experimentation Committee of Kansai Medical University.

### nNOSNT-YFP translocation assay

PC12N cells were plated on poly-L-lysine-coated glass-bottomed 35-mm dishes at a density of 1 × 10^4 ^cells/cm^2 ^and caused to differentiate by 5-day treatment with 50 ng/ml NGF. Translocation of nNOSNT-YFP was examined in the cells essentially as reported previously [22]. Briefly, after a 30-min incubation with test agents, the cells were rinsed with phosphate-buffered saline, fixed with 4% paraformaldehyde in 0.12 M sodium phosphate buffer, pH 7.4, for 20 min at room temperature, and then rinsed with phosphate-buffered saline. Digital images were captured on a Zeiss LSM510 laser-scanning confocal microscope (Oberkochen, Germany). The intensity of nNOSNT-YFP fluorescence was quantified by using ImageJ. To evaluate the translocation of nNOSNT-YFP to the plasma membrane in PC12N cells, we counted the number of cells possessing foci of nNOSNT-YFP on their plasma membrane and expressed this number as a percentage of the total cells examined. More than 40 cells were observed for each datum point, and at least 4 experiments were carried out in each analysis.

### Primary culture

The isolation and primary culture of neurons were prepared from the spinal cord of E13-E15 ddY mice. Briefly, after the pregnant animals had been anesthetized with isoflurane, the spinal cords below the cervical segment were collected from embryos under sterile technique and placed in ice-cold phosphate-buffered saline containing 0.1% glucose. The spinal cords were minced with scissors in 5 ml of DMEM containing 0.5 mg/ml of trypsin and kept at 37°C for 30 min. After incubation, 5 ml of DMEM containing 10% fetal calf serum, 40 ng/ml gentamicin, and 50 μl DNase I were added. The cell pellet was suspended in 5 ml of DMEM containing 10% fetal calf serum and 40 ng/ml gentamicin, filtrated and centrifuged for 5 min at 1000 rpm. After washing the cell pellet twice, the spinal neurons were plated on 35-mm dishes at 1 × 10^5 ^cells/ml. The cells were kept in DMEM containing 10% fetal calf serum, 20 ng/ml NGF, 40 ng/ml gentamicin and 10 μM Ara-C at 37°C in a CO_2 _incubator for 24 h, replaced with fresh media without Ara-C, and cultured for 3–7 days until use.

### Measurement of intracellular free Ca^2+ ^concentration ([Ca^2+^]i) and cAMP content

Changes in [Ca^2+^]_i _in NGF-differentiated PC12 cells, spinal neurons and spinal slices were measured as described previously [21,22]. After the cells had been incubated with 5 μM fura-2acetoxymethyl ester for 30 min in DMEM containing 5% fetal calf serum and 10% horse serum, the fura-2-loaded cells on an inverted fluorescence microscope (Olympus IX-70, Tokyo, Japan) were stimulated with ATP, 2-MeSATP or UTP in HEPES-buffered saline solution (HBS) or in Ca^2+^-free HBS supplemented with 6 mM EGTA at 2–3 ml/min. The cells were excited at 340 and 380 nm and the fluorescence emission signal was monitored by using an Aquacosmos-Ratio imaging system (Hamamatsu Photonics, Hamamatsu, Japan) with a cooled charge-coupled device camera. [Ca^2+^]i was expressed as a ratio of fluorescence emission intensity at 340 and 380 nm. For the measurement of [Ca^2+^]i changes in spinal slices, lumbosacral segments prepared from 7-14-day-old ddY mice were cut using a vibrating blade microtome (VT-1000S; Leica, Nussloch, Germany), and slices were incubated for 2 h in artificial cerebrospinal fluid bubbled with 95% O_2 _and 5% CO_2 _at 37°C. The slices (350-μm thick) thus obtained from lumbar segments L4-L6 were used for [Ca^2+^]i measurements.

For measurement of cAMP contents, NGF-differentiated PC12 cells were incubated for 15 min with test agents in HBS containing 0.5 mM 3-iso-butyl-1-methylxanthine, and intracellular cAMP levels were determined by use of a cAMP radioimmunoassay kit (GE Healthcare, Piscataway, NJ, USA).

### Measurement of NO in spinal slices

Neuropathic pain mode was prepared by left L5 spinal nerve transection of 3-week-old ddY mice and slices were prepared from the lumbar spinal cord of the neuropathic pain model mice 7 days after operation as described previously [20]. After a 2-h incubation of the slices in artificial cerebrospinal fluid containing 1 mM L-NAME bubbled with 95% O_2_/5% CO_2 _at 37°C, slices were incubated in artificial cerebrospinal fluid containing 10 μM DAR-4M AM and 1 mM L-NAME for 2 h at room temperature. The slices were kept in a Krebs solution containing 1 mM L-NAME for more than 1 h after loading, and then were placed in the recording chamber. NO generation in spinal slices was started by replacement of 1 mM L-NAME with 1 mM arginine in a Krebs superfusion buffer equilibrated with 95% O_2_/5% CO_2_. The slices were excited at 540 ± 10 nm and the fluorescence emission signal with a >590-nm long-pass filter was monitored by using the Aquacosmos-Ratio imaging system. Increase in NO formation in the spinal cord was expressed as the ratio of fluorescence intensity of DAR-4M to that prior to treatment.

### Reverse transcriptase-polymerase chain reaction (RT-PCR)

Total RNAs were isolated from NGF-treated or untreated PC12N cells and the spinal cord with TRIzol reagent (Invitrogen-Life Technol., Carlsbad, CA, USA), and the first-strand cDNA was synthesized from 1 μg of total RNA by using a ReverTra Ace-α kit (Toyobo, Osaka, Japan). PCR reactions were carried out in a total 25 μl of PCR buffer containing 1.25 units of GeneTaq DNA polymerase (Nippon Gene, Tokyo, Japan), 1.5 mM MgCl_2_, 200 μM concentration of each deoxynucleoside triphosphate, and 0.4 mM specific oligonucleotide primers defined in Table 1. The PCR conditions consisted of an initial denaturation at 94°C for 3 min, followed by 25 cycles (β-actin) or 40 cycles (the other genes) of amplification (94°C for 1 min, and 65°C for 1 min and 72°C for 1 min), and ending with a final 5-min extension at 72°C. PCR products were separated on 2% agarose gels in Tris-acetate-EDTA buffer, stained with ethidium bromide, visualized under ultraviolet light, and photographed.

**Table 1:** Primer sequences employed for RT-PCR analysis

Gene	Primer sequences (5' to 3')	Product length (bp)
P2X_1 _(forward)	TGATCTGGACTGGCACGTTC	441
P2X_1 _(reverse)	GGTCCTCATGTTCTCCTGCA	
P2X_3 _(forward)	TGGCG TTCTG GGTAT TAAGA TCGG	743
P2X_3 _(reverse)	CAGTG GCCTG GTCAC TGGCG A	
P2X_4 _(forward)	GAGGC ATCAT GGGTA TCCAG ATCAA G	447
P2X_4 _(reverse)	GAGCG GGGTG GAAAT GTAAC TTTAG	
P2X_6 _(forward)	CGATT CACTC TCCAG TCCG	317
P2X_6 _(reverse)	GGTCC TCCAG TAGAA ACCG	
P2Y_2 _(forward)	CGCTT CAACG AGGAC TTCA	589
P2Y_2 _(reverse)	CCATG AGCAC GTAAC AGAC	
P2Y_4 _(forward)	GTTGC CTATG AGCTA TGCAG	518
P2Y_4 _(reverse)	ACCAT GACTG CCGAA CTGAA	
P2Y_6 _(forward)	GGAGA CCTTG CCTGC CGCCT GGTA	410
P2Y_6 _(reverse)	TACCA CGACA GCCAT ACGGG CCGC	
β-actin (forward)	TTCTA CAATG AGCTG CGTGT GGC	456
β-actin (reverse)	CTC(A/G)T AGCTC TTCTC CAGGG AGGA	982
G3PDH (forward)	TGAAGGTCGGTGTGAACGGATTTGGC	
G3PDH (reverse)	CATGTAGGCCATGAGGTCCACCAC	

### Statistics

All data were presented as the mean ± SEM. Statistical analyses of the results were made by using Student's *t *test or the Mann-Whitney U test. EC_50 _values were calculated by use of the computer program for graded responses (ver 1.2) http://chiryo.phar.nagoya-cu.ac.jp/javastat/Graded50-j.htm.

## Results

### Translocation of nNOS from the cytosol to the plasma membrane by ATP

**Figure 1:**
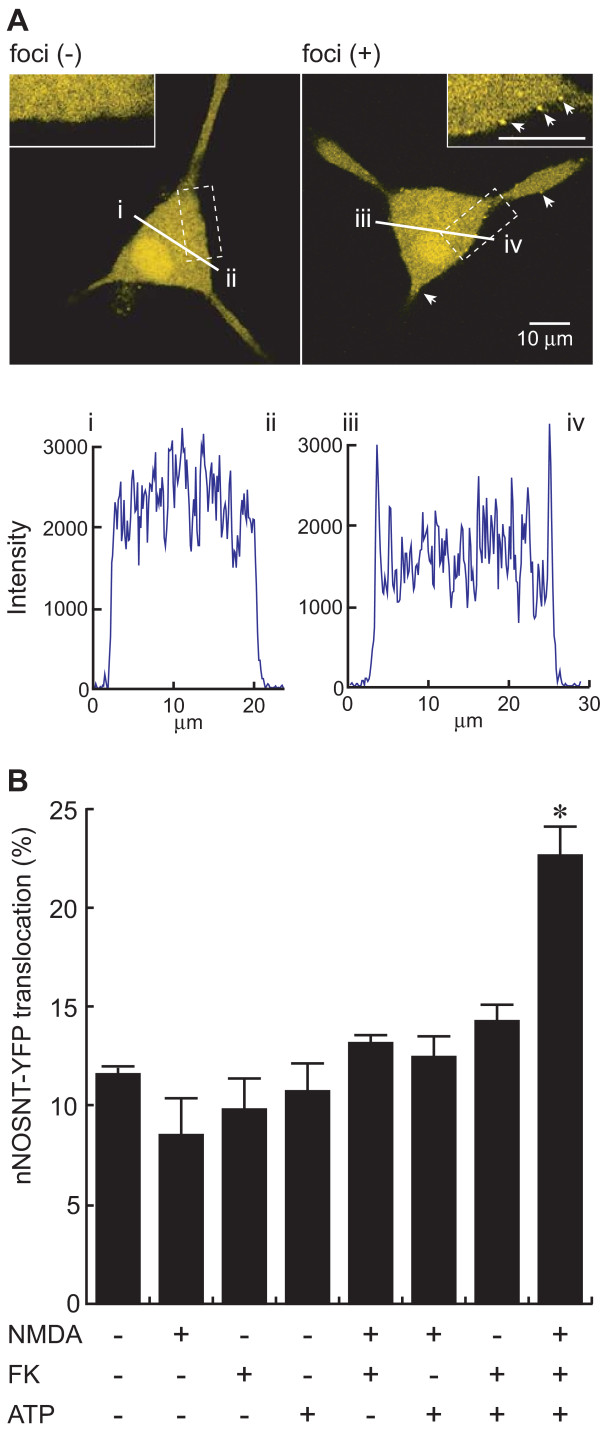
**Translocation of nNOSNT-YFP by ATP in PC12N cells**. **A**. Expression and translocation of nNOSNT-YFP in a PC12N cell. After a 30-min incubation of NGF-differentiated PC12N cells with 100 μM ATP, 100 μM NMDA, and 10 μM forskolin (FK), the cells were fixed; and then fluorescent images of the cells without (left) and with (right) foci were acquired by a confocal microscope. *Insets *in "**A**" depict higher magnification of the cells delineated by rectangles. The fluorescence intensity of nNOSNT-YFP was quantified along the indicated lines by using ImageJ. In these digital images, points "i-ii" and "iii-iv" represent the edges of the cells. **B**. Involvement of ATP in nNOSNT-YFP translocation in PC12N cells. After a 30-min incubation of NGF-differentiated PC12N cells with the indicated combinations of 100 μM ATP, 100 μM NMDA, and/or 10 μM FK, the translocation was calculated as described under "Methods." About 40 cells in a 35-mm glass-bottomed dish were examined in each translocation assay by using a confocal microscope; and the cells showing foci of yellow fluorescence of nNOSNT-YFP on the plasma membrane, as shown in "**A**," were considered to be positive for nNOS translocation. Data are presented as the mean ± SEM of four translocation assays. *p < 0.05 compared with vehicle.

We recently succeeded in visualizing nNOS translocation from the cytosol to the plasma membrane in PC12N cells stably expressing nNOSNT-YFP and demonstrated that the translocation is mediated by activation of PKA and PKC in the presence of NMDA [22]. Since ATP is known to release glutamate from primary afferent fibers and also to act as an excitatory transmitter in the dorsal horn of the spinal cord, we examined whether ATP could cause the translocation of nNOSNT-YFP in PC12N cells. Although the formation of nNOSNT-YFP foci was observed on the plasma membrane of about 10% of the NGF-differentiated PC12N cells, nNOSNT-YFP was exclusively observed in the cytosol of most cells in the microscopic field. When NGF-differentiated PC12N cells were simultaneously treated for 30 min with 100 μM ATP, 100 μM NMDA, and 10 μM forskolin, an adenylate cyclase activator, the number of the cells microscopically showing the foci of yellow fluorescence of nNOSNT-YFP on the plasma membrane apparently increased. Figure 1A presents such cells without and with foci after the treatment. As compared with that along the line "i-ii " the fluorescence intensity along the line "iii-iv" (lower panel in Figure 1A) clearly showed that the foci of nNOSNT-YFP were located on the plasma membrane. When the cells showing the foci were counted as being positive for the nNOSNT-YFP translocation to the plasma membrane, the translocation was significantly enhanced by simultaneous stimulation with 100 μM ATP, 100 μM NMDA, and 10 μM forskolin (22.5 ± 1.64% vs. 11.6 ± 0.40% of the cells without stimulation, *p < 0.05), but not with NMDA, forskolin, and ATP alone or in any other combinations (Figure 1B). These results suggest that ATP would induce the nNOS translocation in PC12N cells in the presence of NMDA and forskolin.

### Involvement of P2 and NMDA receptors in nNOS translocation by ATP

**Figure 2:**
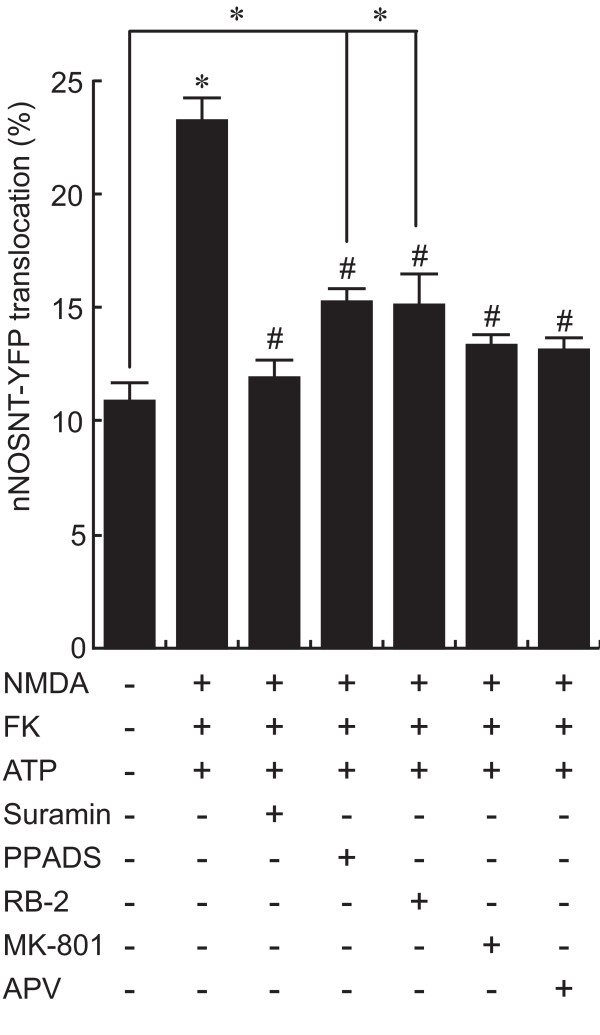
**Involvement of purinergic and NMDA receptors in ATP-induced nNOSNT-YFP translocation in PC12N cells**. After a 30-min incubation of NGF-differentiated PC12N cells without or with 100 μM NMDA, 10 μM forskolin (FK), and 100 μM ATP in the absence and presence of 250 μM suramin, 100 μM PPADS, 100 μM RB-2, 100 μM MK-801 or 100 μM APV, the translocation of nNOSNT-YFP was observed by confocal microscopy and quantified as described in the legend for Figure 1B. Data are presented as the mean ± SEM (n = 4). *p < 0.05 compared with vehicle; ^#^*p *< 0.05 compared with NMDA, FK, and ATP.

To clarify the involvement of activation of purinergic P2 receptors in the enhancement of nNOSNT-YFP translocation by ATP in the presence of NMDA and forskolin, we first examined the effect of P2 receptor antagonists on the translocation in PC12N cells (Figure 2). The non-selective P2 receptor antagonist suramin at 250 μM (11.96 ± 0.82%, ^#^*p *< 0.05) completely abolished the nNOSNT-YFP translocation enhanced by NMDA, forskolin, and ATP (23.19 ± 0.77%, *p < 0.05 compared with control). This enhanced translocation of nNOSNT-YFP was also significantly inhibited by the P2X receptor antagonist PPADS (15.33 ± 0.54%, ^#^*p *< 0.05) and the P2Y receptor antagonist RB-2 (15.11 ± 1.36%, ^#^*p *< 0.05) at 100 μM. However, the inhibitory effects of both PPADS and RB-2 were only partial; and the translocation remained significantly enhanced as compared with that for the vehicle control.

To examine whether the activation of NMDA receptors was involved in the nNOSNT-YFP translocation with ATP in the presence of NMDA and forskolin, we next examined the effect of NMDA receptor antagonists on nNOS translocation in PC12N cells (Figure 2). The increase in the translocation was completely blocked by NMDA receptor antagonists MK-801 and APV. These results demonstrate that the activation of both P2 and NMDA receptors was required for the nNOS translocation by ATP in the presence of NMDA and forskolin.

### Involvement of P2X receptors or P2Y receptors in nNOS translocation by ATP

**Figure 3:**
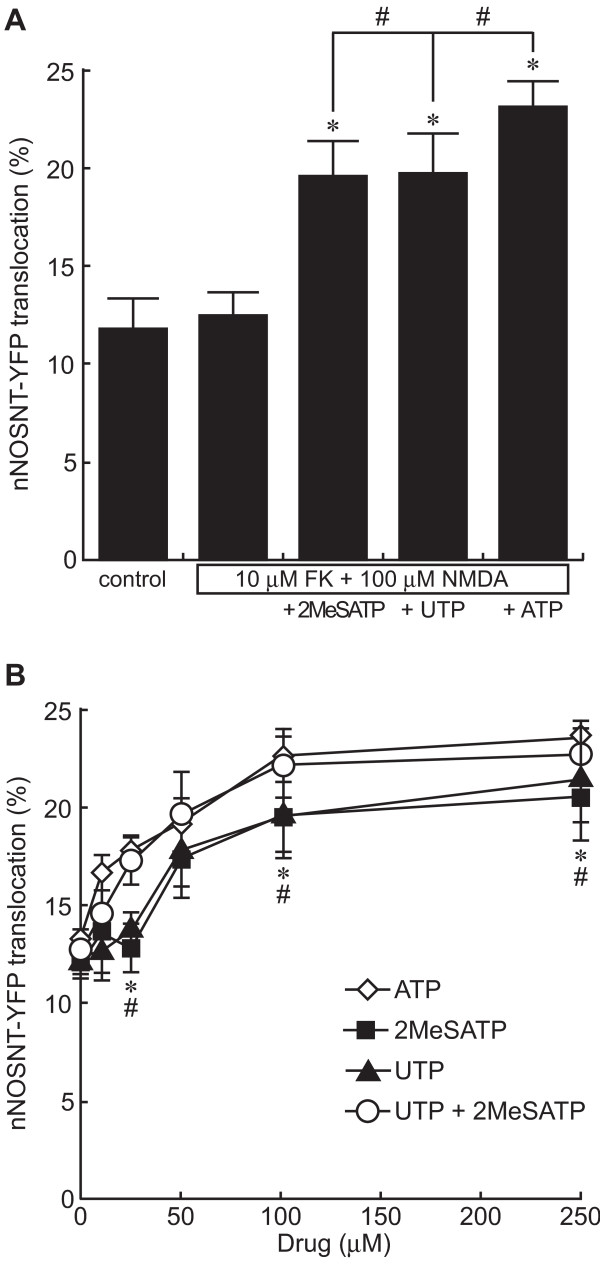
**Effect of P2X and P2Y receptor agonists on nNOSNT-YFP translocation in PC12N cells**. **A**. Stimulation of the translocation of nNOSNT-YFP by P2X and P2Y receptor agonists. After a 30-min incubation of NGF-differentiated PC12N cells without and with 100 μM 2MeSATP, UTP or ATP in the presence of 10 μM forskolin (FK) and 100 μM NMDA, the translocation of nNOSNT-YFP was determined by confocal microscopy as described in the legend for Figure 1B. Data are presented as the mean ± SEM (n = 4). *p < 0.05 compared with vehicle; ^#^*p *< 0.05 compared with NMDA, FK, and ATP. **B**. Concentration dependency of ATP and its analogs for the translocation of nNOSNT-YFP in PC12N cells. NGF-differentiated PC12N cells were incubated for 30 min with the indicated concentrations of ATP (◇), 2-MeSATP (■), UTP (▲) or UTP + 2-MeSATP (○) in the presence of 10 μM FK and 100 μM NMDA. Data are presented as the mean ± SEM (n = 4). *p < 0.05 ATP vs. 2-MeSATP; ^#^*p *< 0.05 ATP vs. UTP.

Conversely we tested the effect of P2 receptor agonists on the translocation of nNOSNT-YFP to the plasma membrane in PC12N cells in the presence of NMDA and forskolin (Figure 3A). The translocation of nNOSNT-YFP was increased from 12.1 ± 0.98% to 19.7 ± 0.88% by 100 μM 2-MeSATP, a P2X receptor agonist, and to 19.6 ± 2.16% by 100 μM UTP, a P2Y receptor agonist. These results suggest that the translocation of nNOSNT-YFP in PC12N cells was induced by the activation of either P2X receptors or P2Y receptors. Figure 3B shows the concentration-response curves of ATP, 2-MeSATP, and UTP for nNOSNT-YFP translocation in the cells. The translocation of nNOSNT-YFP was observed at and above 50 μM ATP and reached a plateau at 100 μM. On the other hand, the translocation of nNOSNT-YFP started to increase with 2-MeSATP or UTP at 50 μM but the potencies of the nNOSNT-YFP translocation by 2-MeSATP and UTP were lower than that by ATP with EC_50 _values of 27 and 25 μM, respectively. However, the co-stimulation with 2-MeSATP and UTP showed almost the same concentration curve for the translocation of nNOSNT-YFP as that obtained with ATP stimulation. Since 2-MeSATP and UTP are the respective agonists of P2X and P2Y receptors, these results demonstrate that the translocation of nNOSNT-YFP to the plasma membrane by ATP was additively mediated by activation of P2X and P2Y receptors.

### Signal transduction coupled to ATP and involved in nNOS translocation

**Figure 4:**
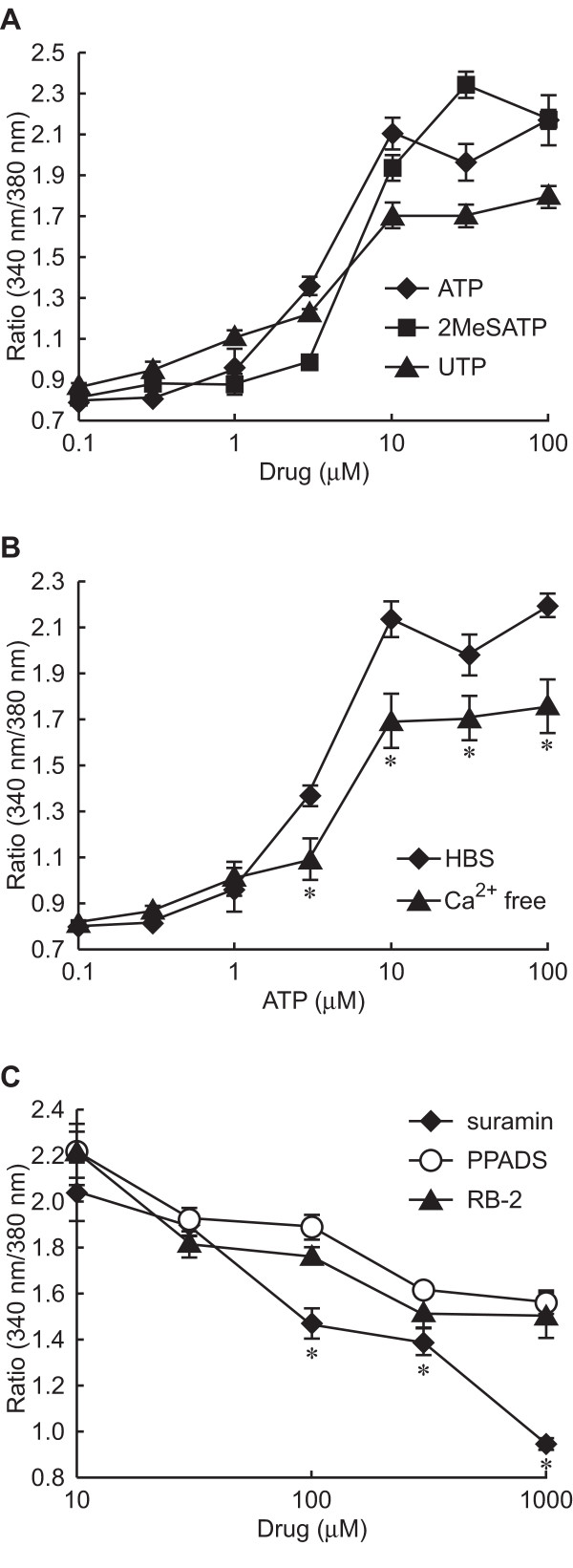
**Effect of ATP and ATP analogues on intracellular Ca^2+ ^concentration ([Ca^2+^]i) in PC12 cells**. **A**. Concentration dependency of [Ca^2+^]i increase elicited by ATP or ATP analogues in NGF-differentiated PC12 cells. Fura-2-loaded PC12 cells were stimulated with the indicated concentrations of ATP (◆), 2-MeSATP (■) or UTP (▲) in HBS, after which the change in [Ca^2+^]i was measured as a fluorescence ratio obtained with excitation at 340 and 380 nm, as described under "Methods." **B**. Effect of extracellular Ca^2+ ^on ATP-induced [Ca^2+^]i increase. Fura-2-loaded PC12 cells were stimulated with the indicated concentrations of ATP in HBS (◆) or in Ca^2+^-free HBS supplemented with 6 mM EGTA (▲). *p < 0.05 compared with HBS. **C**. Effect of P2 receptor antagonists on ATP-induced [Ca^2+^]i increase. Fura-2-loaded PC12 cells were stimulated with 100 μM ATP in the presence of the indicated concentrations of suramin (◆), PPADS (○) or RB-2 (▲). Data are presented as the mean ± SEM (n = 30–60). *p < 0.05 compared with PPADS or RB-2.

Since both P2X and P2Y receptors are known to increase [Ca^2+^]i, to clarify signaling pathways involved in nNOS translocation by ATP, we next examined the increase in [Ca^2+^]i by ATP and its analogues in fura-2-loaded PC12 cells (Figure 4A). ATP increased [Ca^2+^]i in a concentration-dependent manner with an EC_50 _value of 3.5 μM. 2-MeSATP and UTP also increased [Ca^2+^]i, giving EC_50 _values of 11.8 and 2.8 μM, respectively. P2X receptors are ion channels and P2Y receptors are G-protein-coupled receptors. Next we examined the ATP-induced increase in [Ca^2+^]i in Ca^2+^-free perfusion buffer supplemented with 6 mM EGTA, a Ca^2+ ^chelating reagent (Figure 4B). Although ATP could increase the [Ca^2+^]i in the PC12 cells in the presence of EGTA, the peak level of the increase caused by ATP was lower in the presence of EGTA than in the absence of it, and the concentration-response curve with ATP and EGTA was similar to that obtained with UTP. As the P2 receptor antagonists inhibited the translocation of nNOSNT-YFP to the plasma membrane (Figure 2), we next investigated the effect of P2 receptor antagonists on the increase in [Ca^2+^]i caused by 100 μM ATP (Figure 4C). At 1 mM the non-selective P2 receptor antagonist suramin completely suppressed the ATP-induced increase in [Ca^2+^]i, but PPADS and RB-2 at the same concentration only partially reduced it. These results are consistent with the additive effect on nNOS translocation by P2X and P2Y agonists (Figure 3B).

**Figure 5:**
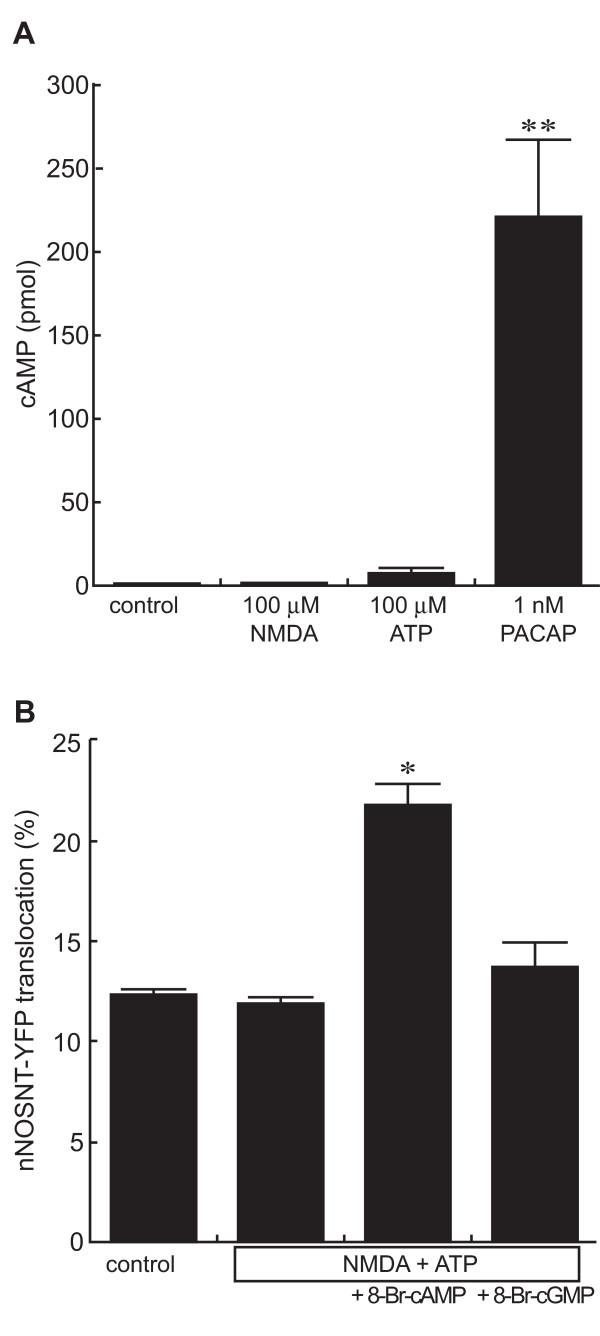
**Involvement of cAMP in ATP-induced nNOSNT-YFP translocation**. **A**. Effect of ATP on cAMP production in NGF-differentiated PC12 cells. The cells (1 × 10^4 ^cells/well) in 24-well plates were incubated for 15 min without or with 100 μM NMDA, 100 μM ATP or 1 nM PACAP in HBS containing 0.5 mM iso-butyl-1-methylxanthine. The cAMP content in the cells was measured by using the cAMP assay kit as described in "Methods." Data are presented as the mean ± SEM (n = 4–7). **p < 0.01 compared with vehicle. **B**. Effect of permeable cAMP and cGMP analogues on nNOSNT-YFP translocation. After a 30-min incubation of NGF-differentiated PC12N cells with vehicle, 500 μM 8-Br-cAMP or 500 μM 8-Br-cGMP in the presence of 10 μM forskolin and 100 μM NMDA, the translocation of nNOSNT-YFP was quantified as described in the legend for Figure 1B. Data are presented as the mean ± SEM (n = 4). *p < 0.05 compared with vehicle.

Since PACAP increases intracellular cAMP levels as well as [Ca^2+^]i via PACAP receptor 1 and induces nNOSNT-YFP translocation in PC12N cells [22], next we measured intracellular cAMP levels 15 min after in the presence of 0.5 mM iso-butyl-1-methylxanthine, a phosphodiesterase inhibitor. Whereas PACAP significantly stimulated the intracellular cAMP production, ATP only slightly increased it; and NMDA did not produce cAMP in the PC12 cells (Figure 5A). Since ATP did not promote the production of cAMP, we next examined the necessity of cAMP production in the translocation of nNOSNT-YFP. We tested the effect of 8-Br-cAMP, a membrane-permeable analog of cAMP, on the translocation of nNOSNT-YFP (Figure 5B). 8-Br-cAMP increased the nNOSNT-YFP translocation to the plasma membrane (21.8 ± 0.98% vs. 11.4 ± 0.55% with vehicle, *p < 0.05); but 8-Br-cGMP, a membrane-permeable analog of cGMP, failed to stimulate it (13.7 ± 1.24%). These results are consistent with our previous finding that the cAMP/PKA pathway is required for the translocation of nNOSNT-YFP in PC12N cells.

### Signaling pathways involved in nNOS translocation by ATP in PC12N cells

**Figure 6:**
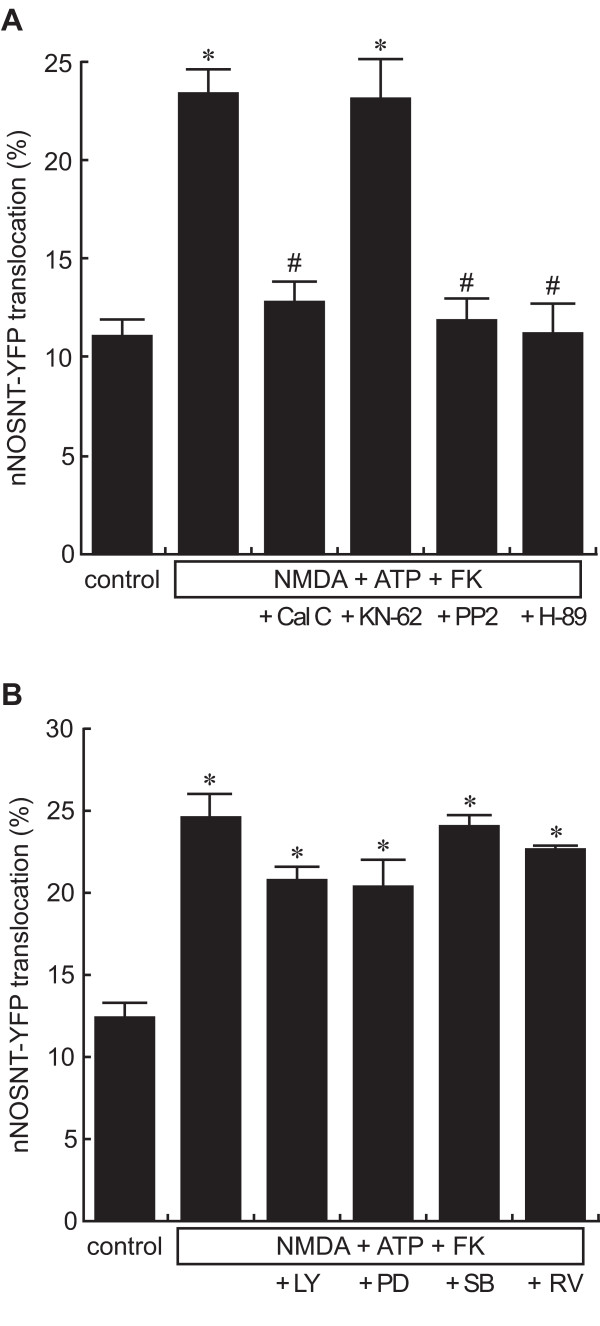
**Signal pathways coupled to the ATP-induced nNOSNT-YFP translocation**. Effect of kinase inhibitors on the translocation of nNOSNT-YFP induced by NMDA, forskolin, and ATP. After a 30-min incubation of NGF-differentiated PC12N cells with vehicle or with 100 μM NMDA, 100 μM ATP, and 10 μM forskolin (FK) in the presence of 0.1 μM calphostin C (Cal C), 1 μM KN-62, 1 μM PP2, 50 nM H-89, 25 μM LY294002 (LY), 25 μM PD98059 (PD), 20 μM SB203580 (SB) or 25 μM roscovitine (RV), the translocation of nNOSNT-YFP was observed by confocal microscopy and quantified as described in the legend for Figure 1B. Data are presented as the mean ± SEM (n = 4). *p < 0.05 compared with vehicle; ^#^*p *< 0.05 compared with NMDA, ATP, and FK.

To clarify the signal pathways involved in nNOSNT-YFP translocation induced by ATP in the presence of NMDA and forskolin, we examined the effects of various inhibitors of protein kinases that can be activated by ATP. Similar to the other sets of translocation experiments, the translocation of nNOSNT-YFP was induced with 100 μM ATP, 100 μM NMDA, and 10 μM forskolin. The PKC inhibitor calphostin C (12.6 ± 0.90%, ^#^*p *< 0.05), the Src inhibitor PP2 (11.8 ± 0.93%, ^#^*p *< 0.05), and the PKA inhibitor H-89 (11.1 ± 1.48%, ^#^*p *< 0.05) inhibited the translocation of nNOSNT-YFP; whereas KN-62, an inhibitor of Ca^2+^/calmodulin-dependent protein kinase II failed to inhibit it (Figure 6A).

To further clarify the signal pathways involved in the translocation in PC12N cells, we examined the effect of other kinase inhibitors, LY294002 (phosphatidylinositol 3-kinase), PD98059 (extracellular signal-regulated kinase), SB203580 (p38 mitogen-activated protein kinase), and roscovitine (cyclin-dependent kinase 5) on the translocation of nNOSNT-YFP induced by ATP, NMDA, and forskolin (Figure 6B). None of them significantly attenuated the translocation. These results confirmed our recent findings [22], demonstrating that nNOS translocation is mediated by activation of PKA, PKC, and Src kinase in PC12 cells.

### Expression of P2X and P2Y receptors in PC12N cells and spinal cord

**Figure 7:**
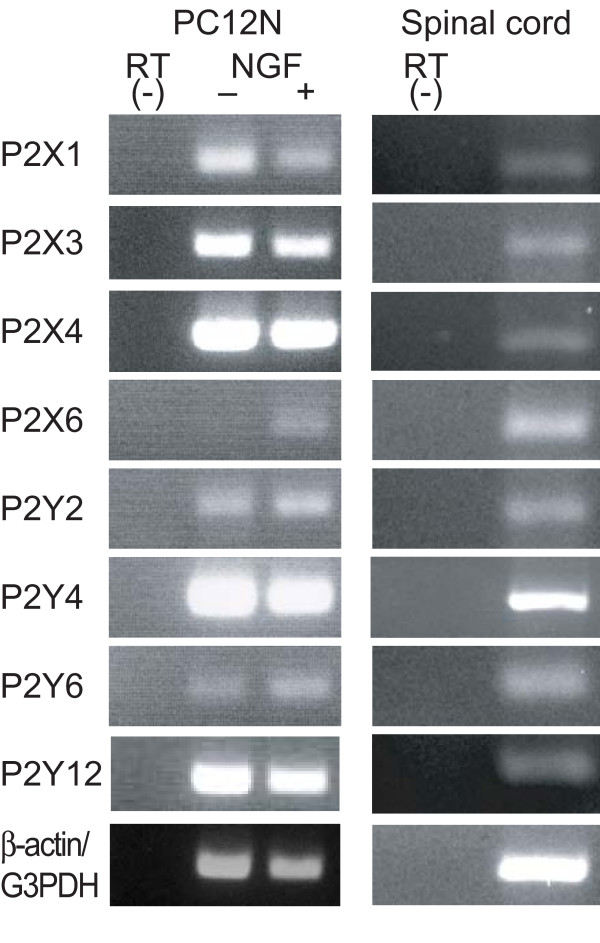
**mRNA expression of purinergic receptors in PC12N cells and spinal cord**. PC12N cells were incubated without (-) or with (+) 50 ng/ml NGF for 5 days. Total RNA was extracted from the cells and the spinal cord and subjected to RT-PCR using the primers shown in Table 1. PCR products were analyzed by 2% agarose gel electrophoresis and stained with ethidium bromide. β-Actin and G3PDH were used for PC12N cells and for the spinal cord as the control and total RNA without RT was used as negative control.

To date 7 ionotropic P2X receptors and 8 G-protein-coupled metabotropic P2Y receptors have been cloned, and most of them are expressed on primary afferent neurons or spinal dorsal horn neurons. So next we examined the expression of P2X and P2Y subtypes in NGF-undifferentiated or differentiated PC12N cells and the spinal cord by RT-PCR (Figure 7). In undifferentiated PC12 cells, bands corresponding to mRNAs for P2X_1_, P2X_3_, P2X_4 _receptors were detected with the expected sizes. In NGF-differentiated PC12N cells, the same transcripts were present; and, in addition, P2X_6 _receptor mRNA was detected. P2Y_2_, P2Y_4_, P2Y_6_, and P2Y_12 _receptor mRNAs were also present in both undifferentiated and NGF-differentiated PC12N cells. These results are consistent with those of a previous study [26]. These transcripts of P2X and P2Y receptors were detected in the spinal cord. These results suggest that ATP may induce nNOS translocation in the spinal cord as well as in PC12N cells.

### Characterization of [Ca^2+^]i responses to ATP and UTP in cultured spinal neurons

**Figure 8:**
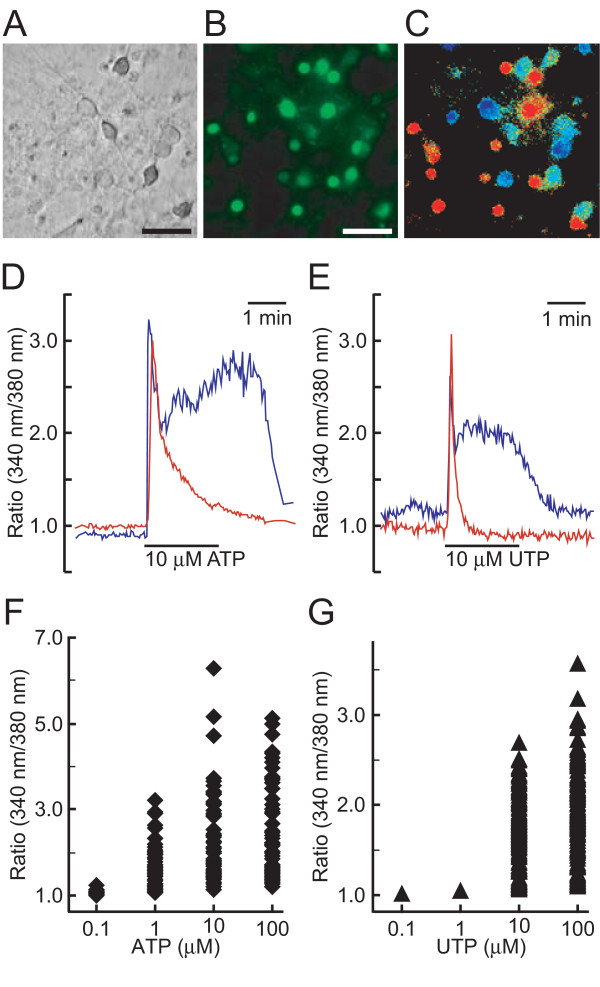
**Effect of ATP and UTP on intracellular Ca^2+ ^concentration ([Ca^2+^]i) in spinal neurons**. **A-C**. Increase in [Ca^2+^]i by ATP in spinal neurons. Bright-field photomicrograph of cultured spinal neurons (**A**), fluorescence image of fura-2-loaded neurons excited at 340 nm (**B**), and ATP (10 μM)-evoked [Ca^2+^]i changes in spinal neurons shown in pseudocolor as the ratio of fura-2 fluorescence intensity at 340/380 nm (**C**). Scale bars, 50 μm. **D, E**. Representative traces of [Ca^2+^]i changes in single spinal neurons by ATP and UTP. Spinal neurons in the same field showed phasic (red) and tonic (blue) responses by 10 μM ATP (**D**) and 10 μM UTP (**E**). **F, G**. Distribution of [Ca^2+^]i responses of individual spinal neurons to ATP and UTP. Fura-2-loaded spinal neurons were sequentially stimulated with the indicated concentrations of ATP (◆) and UTP (▲) in HBS, after which the peak levels of [Ca^2+^]i in single neurons were plotted at each concentration. Total 177 and 143 cells of 3 independent primary cultures were measured for ATP and UTP, respectively, and about 90% of the cells responded to ATP and UTP at 100 μM.

**Figure 9:**
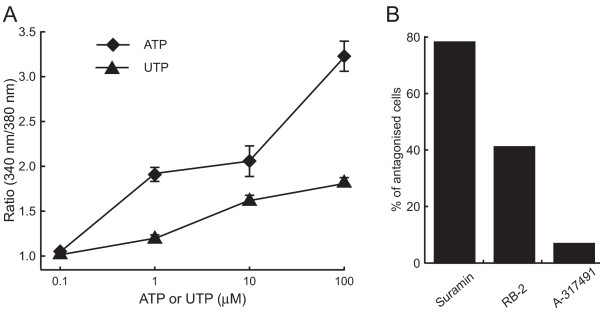
**Characterization of [Ca^2+^]i responses to ATP and UTP in cultured spinal neurons**. **A**. Concentration dependency of [Ca^2+^]i increase elicited by ATP (◆) or UTP (▲) in primary cultured spinal neurons. **B**. Effect of P2 antagonists on ATP-evoked [Ca^2+^]i increase in spinal neurons. Fura-2-loaded spinal neurons were sequentially stimulated with 1 μM ATP, 1 μM ATP with 100 μM suramin, 100 μM RB-2 or 10 μM A-317491, and then 1 μM ATP in HBS at more than 30-min periods for washing between stimulation, fluorescence images were taken at 5 sec intervals. Neurons that the ATP-evoked [Ca^2+^]i increase was considerably reduced were taken as antagonized and expressed as % of ATP-responsive cells (54 for suramin, 117 for RB-2, and 118 for A-317491).

We next examined whether ATP and UTP could increase [Ca^2+^]i in primary cultured spinal neurons (Figure 8A). [Ca^2+^]i increase of the fura-2-loaded neurons was observed by the treatment with 1 μM ATP (Figure 8B and 8C). The time course and magnitude of [Ca^2+^]i increase varied from cell to cell in primary cultured spinal neurons. Representative traces of [Ca^2+^]i change from a single cell showed both phasic and tonic patterns by either ATP (Figure 8D) or UTP (Figure 8E). The peak level of [Ca^2+^]i increase evoked by ATP and UTP also varied among cells (Figure 8F and 8G) and about 10% of the cells did not respond to ATP and UTP at 100 μM. Whereas both ATP and UTP increased [Ca^2+^]i in spinal neurons in a concentration-dependent manner up to 100 μM, ATP was more potent in elevating [Ca^2+^]i than UTP (Figure 9A). When we examined the effect of P2 receptor antagonists on ATP-evoked [Ca^2+^]i increase in spinal neurons, A-317491, a selective P2X_3_/P2X_2/3 _antagonist, RB-2, and suramin reduced the increase in 6.8, 41, and 78% of the cells responsive to 1 μM ATP, respectively (Figure 9B).

**Figure 10:**
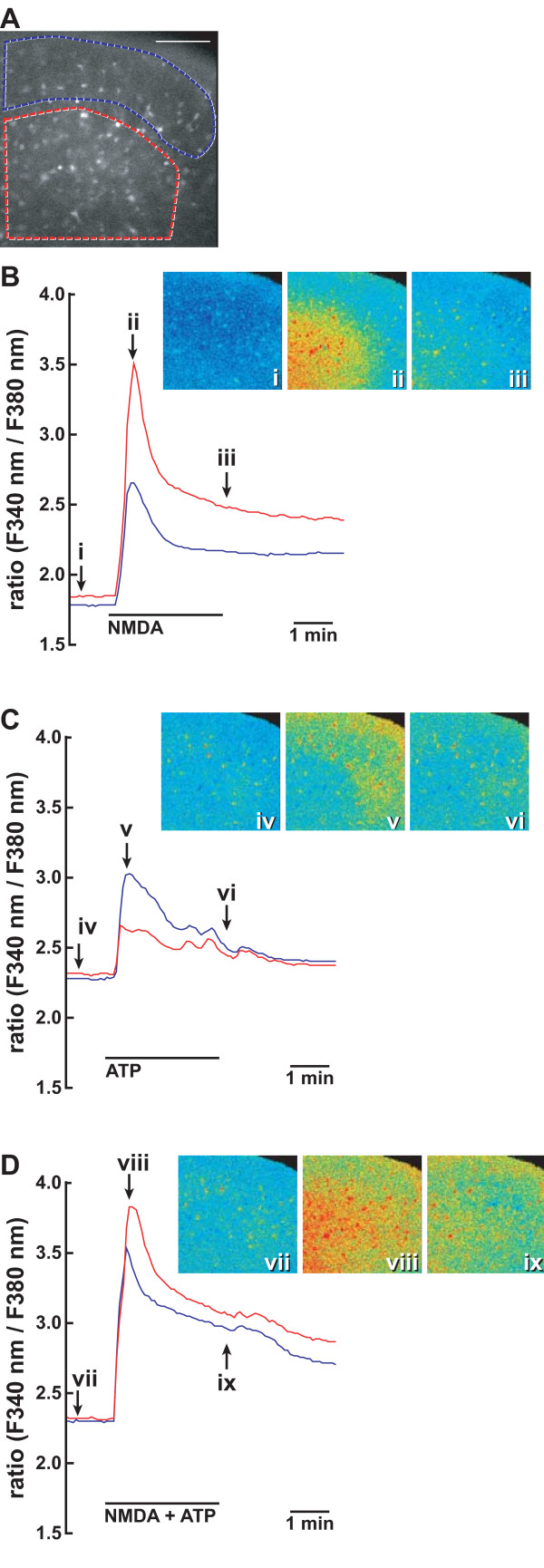
**Enhancement of NMDA-evoked [Ca^2+^]i increase by ATP in the spinal cord**. **A**. Fluorescence image of a transverse section of fura-2-loaded L5 spinal cord excited at 340 nm. Areas surrounded by blue and red lines depict the regions to monitor fluorescence images of the dorsal horn. **B-D **represent typical traces of two regions showing the enhancement and prolongation of NMDA-evoked [Ca^2+^]i increase by ATP. The slice was serially stimulated by 100 μM NMDA (**B**), 100 μM ATP (**C**), and 100 μM NMDA and 100 μM ATP (**D**). Between stimulation, the spinal slice was perfused by the Krebs solution for more than 30 min. [Ca^2+^]i changes in spinal slices were fluorometrically monitored at 5-s intervals. [Ca^2+^]i changes are expressed as a ratio of fluorescence emission intensity excited at 340/380 nm. Fluorescence images (**i**)-(**ix**) are shown in pseudocolour as ratio images at the indicated point on [Ca^2+^]i traces (**B-D**). Similar results were obtained with 10 slices prepared from 5 mice. Bar = 100 μm.

To further clarify whether nNOS was activated by ATP in the spinal cord, we prepared 10 spinal slices from 5 wild-type mice and measured [Ca^2+^]i changes in fura-2-loaded slices in situ (Figure 10A). Spinal slices were serially stimulated with 100 μM NMDA, 100 μM ATP, and 100 μM NMDA+100 μM ATP. To prevent the refractoriness of [Ca^2+^]i responses to repeated stimulation by ATP, we stimulated the slices at more than 30-min intervals and confirmed the [Ca^2+^]i increase caused by repeated stimulation with 100 μM ATP (data not shown). Figure 10B–D shows representative traces of the [Ca^2+^]i change in the superficial and deeper layers of the spinal cord. NMDA alone induced a rapid and transient [Ca^2+^]i increase and the increase was higher in the deeper layer than in the superficial layer (Figure 10B). On the other hand, ATP alone also increased [Ca^2+^]i in the superficial layer rather than in the deeper layer (Figure 10C). When the spinal slice was simultaneously stimulated by NMDA and ATP, the [Ca^2+^]i increase was enhanced and prolonged both in the superficial layer and deeper layers (Figure 10D).

### Effect on P2X3 antagonist on NO formation in the spinal cord of neuropathic pain model mice

**Figure 11:**
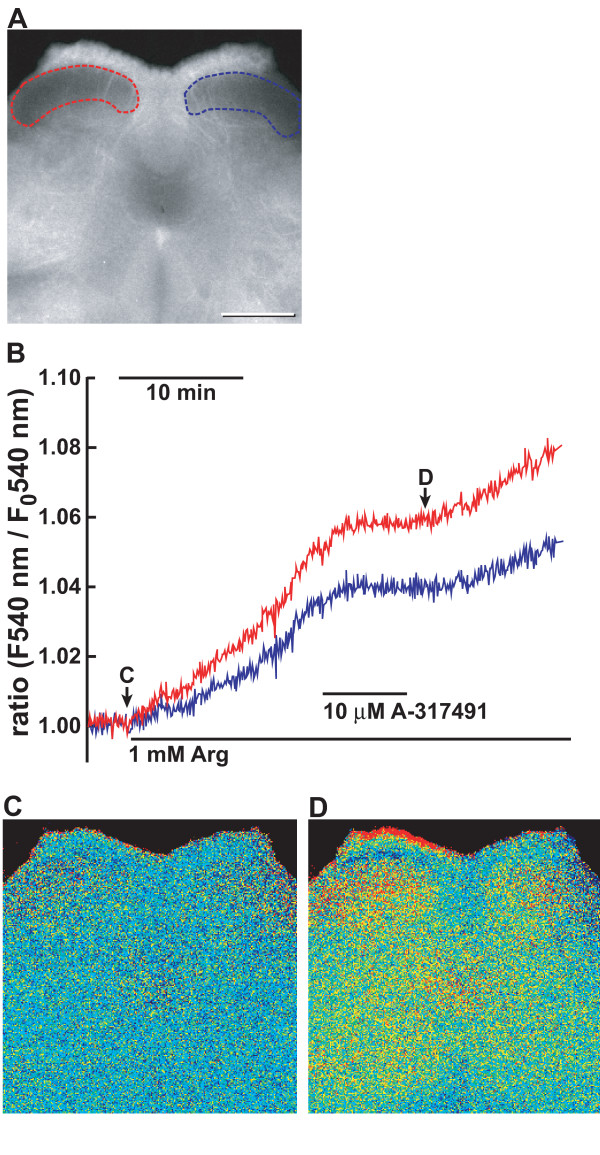
**Effect of A-317491 on NO production in the spinal cord 7 days after L5 spinal nerve transection**. **A**. Fluorescence image of a transverse section of DRA-4M-loaded L5 spinal cord excited at 540 nm. Areas surrounded by red and blue lines depict the regions to monitor fluorescence of the dorsal horn in the sides ipsilateral and contralateral to L5 spinal nerve transection. **B-D**. Inhibition of NO formation by A-317491 in the dorsal horn of neuropathic pain model mice. Spinal slices were prepared from the L5 segment of neuropathic pain model mice 7 days after L5 spinal nerve transection. Fluorescence imaging for NO was obtained in the spinal slice loaded with DAR-4M and images were taken at 5-s intervals as described in "Methods". **B **shows representative traces of inhibition NO formation by A-317491 in the spinal slice prepared from neuropathic pain model mice. Underlines indicate the presence of 10 μM A-317491 and 1 mM L-arginine in the Krebs buffer. **C, D**. Fluorescence images of DAR-4M-loaded spinal cord at (**C**, *an arrow*) and 25 min after **(D) **the replacement of 1 mM L-NAME with 1 mM L-arginine. Fluorescence images are shown in pseudocolour as ratio images on the basis of the initial intensity at the start of measurement. Similar results were obtained with 5 slices of 5 animals. Bar = 500 μm.

The purinergic receptors in the spinal cord are shown to be involved in neuropathic pain. We previously showed that the increase in nNOS activity in the superficial dorsal horn of the spinal cord reflects a neuropathic pain state even 1 week after nerve injury [20]. To study whether ATP was involved in neuropathic pain by nNOS activation, we directly examined the effect of A-317491 on nNOS activation in neuropathic pain model mice using the fluorescent NO indicator DAR-4M (Figure 11A). To avoid endogenous NO production and depletion of L-arginine, a substrate of NOS, in the spinal cord during preparation and loading DRA-4M, 1 mM L-NAME was added to the solution. When L-NAME in a Krebs solution was replaced by 1 mM L-arginine, the fluorescence intensity gradually increased over 30 min in the dorsal horn in the lumbar spinal slice prepared from mice 7 days after left L5 spinal nerve transection. The increase was more prominent in the superficial layer of the ipsilateral side than of the contralateral side (Figure 11B and 11C). These increases in the ipsilateral and contralateral sides were blocked by the addition of A-317491 and resumed by its washout (Figure 11D).

## Discussion

### nNOS translocation by ATP

NO is well known to be implicated in nociceptive processing in the spinal cord [17]. Based on the results of a study using mice lacking the PACAP gene [21], we previously suggested that PACAP is a key molecule of pain hypersensitivity that acts by promoting the functional coupling of nNOS to NMDA receptors. To address this suggestion, we recently established a fluorescence imaging system for examining nNOS translocation in PC12N cells and demonstrated that the synergism of PACAP and NMDA was critical for the translocation and activation of nNOS through PKA, PKC, and Src kinase via PACAP receptor 1 and NMDA receptors [22]. Purinergic signaling is involved in long-term inflammatory and neuropathic pain as well as in acute pain [1,2] and ATP increases the intracellular Ca^2+ ^level in differentiated PC12 cells [26]. Different from the synergism of PACAP and NMDA, ATP and NMDA could not translocate nNOSNT-YFP in PC12N cells (Figure 1). This can be explained by the difference in the ability of cAMP formation between PACAP and ATP (Figure 5A). Therefore nNOS was translocated by ATP and NMDA in the presence of forskolin (Figure 1B) or the membrane-permeable cAMP analogue 8-Br-cAMP (Figure 5B). As observed in our recent study [22], nNOSNT-YFP was localized to the membrane in 10–12% of the cells before stimulation and maximally in 25–30% of the cells after the stimulation with 100 μM NMDA and 1 nM PACAP. Since nNOSNT-YFP was over-expressed in PC12N cells, the reason that the maximum translocation was at most 25–30% of the cells might be ascribed to the nature of nNOS translocation by forming a tertiary complex with endogenous PSD-95 and NMDA receptors on the membrane. In fact, a nNOSNT-YFP mutant lacking the β-finger of the PDZ domain of nNOS was scarcely localized to the membrane before stimulation; and the translocation to the membrane was not increased by stimulation with NMDA and PACAP. Furthermore, NO formation by PACAP and NMDA was markedly attenuated in PC12N cells, probably because over-expressed nNOSNT-YFP competed with endogenous nNOS for forming the tertiary complex on the membrane. Consistent with our recent findings [22], the inhibitors of PKA, PKC, and Src kinase blocked the enhancement of nNOSNT-YFP translocation by ATP, forskolin, and NMDA (Figure 6A). Although P2X and P2Y receptors activate several signaling pathways, the inhibitors of other kinases failed to block the translocation of nNOSNT-YFP (Figure 6B). The present study extended our recent studies by use of the fluorescence imaging system and confirmed that the activation of Ca^2+^/PKC and cAMP/PKA pathways is necessary and sufficient for nNOS translocation in the presence of NMDA.

### Characterization of purinergic receptors involved in nNOS translocation

ATP acts via various subtypes of ionotropic P2X receptors and/or metabotropic P2Y receptors. The translocation of nNOSNT-YFP by ATP was completely blocked by the non-selective P2 receptor antagonist suramin and significantly reduced by the P2X antagonist PPADS or the P2Y antagonist RB-2 (Figure 2). Conversely, 2-MeSATP, a P2X receptor agonist or UTP, a P2Y_2 _and P2Y_4 _receptor agonist, stimulated the translocation of nNOSNT-YFP to the plasma membrane (Figure 3A). However, the increase in the translocation of nNOSNT-YFP by 2-MeSATP or UTP was not as great as that obtained with ATP itself. Co-stimulation with 2-MeSATP and UTP increased the translocation of nNOSNT-YFP in a concentration-dependent manner, with the increase being comparable to that obtained with ATP and with similar EC_50 _values (Figure 3B). ATP, 2-MeSATP, and UTP induced the increase in [Ca^2+^]i with EC_50 _values of 3.5, 10.8, and 2.84 μM, respectively, in PC12N cells (Figure 4A); and ATP could increase the [Ca^2+^]i in the absence of extracellular Ca^2+^(Figure 4B), demonstrating that P2Y receptors as well as P2X receptors were involved in the increase in the [Ca^2+^]i of PC12N cells. Furthermore, it is intriguing that, whereas the non-selective P2 receptor antagonist suramin fully suppressed the ATP-induced increase in [Ca^2+^]i, PPADS and RB-2 reduced it only partially (Figure 4C). Consistent with the results of a previous study [26], P2X_1_, P2X_3_, and P2X_4 _mRNAs were detected by RT-PCR in both undifferentiated and NGF-differentiated PC12 cells; and P2X_6 _mRNA was detected in NGF-differentiated PC12 cells (Figure 7). Since P2X_2 _receptors were cloned from NGF-differentiated PC12 cells [27], P2X receptors except for P2X_7 _are present in NGF-differentiated PC12 cells. As for metabotropic P2Y receptors, besides P2Y_12 _receptors that couple to Gi/o, the other P2Y receptors P2Y_1_, P2Y_2_, P2Y_4_, P2Y_6_, and P2Y_11 _are coupled to the Gq_/11 _proteins, leading to stimulation of phosphoinositide metabolism, release of intracellular Ca^2+ ^stores and activation of PKC. P2Y_2_, P2Y_4_, and P2Y_6 _mRNAs were found in both undifferentiated and NGF-differentiated PC12 cells. Since the P2Y_6 _receptor was not activated by UTP, ATP and UTP may act on P2Y_2 _and P2Y_4 _receptors in NGF-differentiated PC12N cells. Arslan *et al*. extensively characterized Ca^2+ ^responses in PC12 cells and suggested that P2X_2_/P2X_2b_, P2X_4_, and P2Y_2 _receptors contribute to the ATP-induced [Ca^2+^]i increase in NGF-differentiated PC12 cells [26]. Thus the presence of multiple P2X and P2Y receptors in PC12 cells suggests that several P2 receptor subtypes are involved in the increase in [Ca^2+^]i and nNOS translocation and that the effects of ATP on them seem to be additive.

Like PC12 cells, many P2X and P2Y receptors mRNAs were detected by RT-PCR in the spinal cord (Figure 7). To clarify the involvement of P2 receptors in nNOS translocation in the spinal cord, we examined the effect of ATP and UTP on [Ca^2+^]i in primary cultured spinal neurons. While ATP and UTP increased the magnitude of [Ca^2+^]i levels in a concentration-dependent manner, ATP was more potent than UTP (Figure 9A), suggesting that both P2X and P2Y receptors are involved in [Ca^2+^]i changes by ATP in the spinal cord. This notion was supported by the effect of antagonists on the ATP-evoked [Ca^2+^]i responses (Figure 9B). While the P2Y receptor antagonist RB-2 inhibited 41%, the non-selective P2 receptor antagonist suramin reduced the increase in 78% of the cells responsive to 1 μM ATP. Interestingly, A-317491, a selective P2X_3 _antagonist, reduced only 6.8% of the ATP-responsive cells. These results are consistent with the previous studies that P2X_3 _receptors are expressed in DRG neurons and are transported to the central process in the spinal cord [28,29].

### Significance of NO formation by ATP in nociceptive transmission

There is growing evidence that purinergic receptors in the central and peripheral nervous systems are involved in modification of pain sensation [1,2]. NO production following activation of P2 receptors has been shown to exhibit both anti-nociceptive and pro-nociceptive effects. The present study showed that ATP increased the translocation of nNOSNT-YFP in PC12N cells in the presence of NMDA and forskolin, suggesting the generalization that the simultaneous activation of PKA, PKC, and Src-family kinase is essential for the translocation of nNOSNT-YFP to the membrane. Although mechanisms of synergism of ATP and NMDA for nNOS translocation (Figure 2) remain to be clarified, the [Ca^2+^]i increase was enhanced and prolonged by simultaneous stimulation of NMDA and ATP both in the superficial layer and deeper layers of the spinal cord *in situ *(Figure 10B–D) In behavioral studies, platelet-activating factor-evoked tactile allodynia is mediated by ATP and the subsequent NMDA and NO cascade through capsaicin-sensitive fibers [30]. PPADS reduces pain-related behaviors by inhibiting the increased activity of the NO/NOS system in a neuropathic pain model [31] Inoue's group recently showed that mice lacking all isoforms of NOS displayed a reduction in nerve injury-induced neuropathic pain and exhibited a reduction in microglia activation in the spinal cord [32]. We previously showed that the increase in nNOS activity in the superficial dorsal horn of the spinal cord reflects a neuropathic pain state even 1 week after nerve injury [20] and that this nNOS activation may be reversibly regulated by the translocation of nNOS from the cytosol to the plasma membrane in the presence of NMDA and PACAP [21]. Consistent with our previous study [20], NO formation in the dorsal horn was more prominent in the ipsilateral side to L5-spinal nerve transection 7 days after operation and this NO formation was markedly blocked by A-317491 (Figure 11). Thus the present study also demonstrates that ATP might be involved in long-term neuropathic pain by promoting the functional coupling of nNOS to NMDA receptors under conditions in which neuromodulators such as prostaglandin E_2 _that couple to the cAMP/PKA pathway are released by continuous noxious inputs from the periphery. Since the stimulation of a single neuron or glia may activate multiple networks, a concomitant stimulation of facilitatory and inhibitory circuits as a result of ATP release is possible.

## Conclusion

In the present study, we demonstrated that ATP could translocate nNOS from the cytosol to the plasma membrane in the presence of NMDA and forskolin by using a fluorescence imaging system. Moreover, the translocation of nNOSNT-YFP to the plasma membrane by ATP was additively mediated by PKC via activation of P2X and P2Y receptors. In a neuropathic pain model, nNOS was in an activated state, which was blocked by the P2X_3_/P2X_2/3_antagonist. The present study suggests that nNOS translocation may be an action mechanism of ATP in nocieptive processing in the spinal cord.

## Competing interests

The authors declare that they have no competing interests.

## Authors' contributions

TO participated in data acquisition of PC12N cells, the statistical analysis, and drafted the manuscript. SM participated in data acquisition of the spinal cord, data analysis and figure preparation. SI is a corresponding author of the manuscript and participated in the design of experiments and data analysis. All authors read and approved the final manuscript.
